# The Effect of Premedication on the Incidence of Gastroesophageal Reflux in 270 Dogs Undergoing General Anesthesia

**DOI:** 10.3390/ani12192667

**Published:** 2022-10-04

**Authors:** Eugenia S. Flouraki, Ioannis Savvas, George Kazakos, Tilemahos Anagnostou, Dimitrios Raptopoulos

**Affiliations:** 1 Surgery Clinic, Department of Veterinary Medicine, School of Health Science, University of Thessaly, 224 Trikalon Str., 43100 Karditsa, Greece; 2 Anaesthesiology and Intensive Care Unit, School of Veterinary Medicine, Aristotle University of Thessaloniki, 54627 Thessaloniki, Greece

**Keywords:** gastroesophageal reflux, premedication, dog, anesthesia, pH, GOR

## Abstract

**Simple Summary:**

Gastroesophageal reflux (GOR) in anesthetized dogs has been extensively studied throughout the last 40 years. However, the factors affecting the incidence of GOR are still in debate while it is widely accepted that GOR is a multifactorial incidence. The aim of the study is to evaluate the effect of three preanesthetic agents (dexmedetomidine, acepromazine, midazolam), combined with three different opioids (morphine, pethidine, butorphanol), commonly used in small animal anesthesia, on the incidence of reflux in anesthetized dogs. Two hundred and seventy dogs were allocated into nine different groups in accordance to the premedication administered. All dogs underwent non-intrathoracic, non-intrabdominal elective surgeries or invasive diagnostic procedures, while the pH of the esophagus was measured with the use of a pH-meter electrode during the procedure. A detection of esophageal pH below 4 and above 7.5 was consider to be GOR. The study outcome suggested that the addition of opioids in premedication enhanced the incidence of GOR, within the reported values when compared to literature data. No differences were observed among the groups in which the three different opioids were used as far as the incidence of GOR was concerned. Nonetheless, castrations resulted in an increased incidence of GOR when compared to invasive diagnostic procedures.

**Abstract:**

The aim of this prospective, non-randomized study was to evaluate the effect of nine different premedication medications on the incidence of gastroesophageal reflux (GOR) in anesthetized dogs. Two hundred and seventy dogs undergoing non-intrathoracic, non-intrabdominal elective surgeries or invasive diagnostic procedures were included in the study, and were allocated into nine groups (30 dogs/group) defined by the type of premedication administered. Premedication consisted of dexmedetomidine with either morphine, pethidine or butorphanol, acepromazine with either one of the three opioids or midazolam with one of the above-mentioned opioids. Anesthesia was induced with propofol and maintained with isoflurane in oxygen. Esophageal pH was measured with the use of a pH-meter electrode and a pH-value less than 4 and over 7.5 was considered to be GOR. The study revealed that 119/270 (44.1%) dogs experienced a reflux episode during anesthesia. The incidence of reflux did not differ among groups (*p* = 0.117). In group AB the dogs refluxed within 10 min of the beginning of pH-measurements, in comparison with group DB in which dogs refluxed within 30 min (*p* = 0.029). Invasive diagnostic procedures had a lower incidence of GOR in comparison to castrations (*p* = 0.09). The outcome of the study suggests that none of the opioids used increased the incidence of GOR in anesthetized dogs.

## 1. Introduction

Gastroesophageal reflux (GOR) is defined as the reverse ebb of stomach contents through the lower esophageal sphincter (LES) towards the esophageal lumen [1]. The most common risk factor influencing the incidence of reflux is a reduction in barrier pressure observed during anesthesia [2,3,4,5,6]. Although GOR is usually not observed or routinely monitored during anesthesia the consequences may be detrimental. In veterinary medicine, it has been reported that GOR during anesthesia may result in esophagitis, esophageal stenosis, esophageal rapture, rhinitis or aspiration pneumonia, which may even lead to death or euthanasia of the patient [7,8,9,10,11,12]. GOR during anesthesia is influenced by many factors included preoperative fasting, type of food administered, premedication sedatives, opioids, anesthetics, type of surgery, recumbency, duration of anesthesia, age [2,3,12,13,14,15,16,17,18,19,20,21,22,23,24,25,26,27,28].

Several drugs widely used for premedication or anesthesia have been evaluated concerning their effect on the incidence of reflux. Sedatives or tranquillizers, such as acepromazine, xylazine, diazepam or midazolam, and opioids, like morphine and pethidine, have been found to decrease LES pressure predisposing to GOR [15,25,26,27,28,29]. The mechanism of action of a-2 agonists is not fully clarified; however, they are supposed to reduce LES pressure [3,30]. Although, acepromazine has been known to decrease LES pressure [17], when used as a sole premedication agent the incidence of reflux remained low (4.8%) [31].

The use of pethidine as a sole preanesthetic agent has been correlated with 55% reduction in absolute risk of developing GOR [28]. On the other hand, premedication with morphine has been associated with a higher risk of GOR [19,27]. The use of butorphanol and midazolam has not been widely evaluated concerning their effects on the incidence of reflux in dogs.

The aim of this study was to investigate the effect of administration of three different opioids and three different sedatives used for premedication and the effect of the type of surgical stimulation on the incidence of reflux, in anesthetized dogs. We hypothesized that morphine in combination with dexmedetomidine would increase the incidence of GOR, whilst the combination of the three different sedatives with pethidine would be associated with a lower risk of GOR. The midazolam groups were expected to have a higher incidence of reflux due to the increased dosage of propofol and isoflurane presumably needed to maintain an adequate depth of anesthesia. Orthopedic surgeries were expected to have a higher incidence of reflux, while invasive diagnostic procedures were expected to have a lower incidence of GOR.

## 2. Materials and Methods

### 2.1. Study Design

This study was approved by the Institution’s Ethical Committee (protocol ID 452/24-2-2000) and the owners’ written informed consent. A total of 270 dogs undergoing scheduled elective surgeries or invasive diagnostic procedures were included in this study. Inclusion criteria were dogs aged from 1 to 11 years with body weight from 4 to 50 kg that were scheduled for non-intrabdominal and non-intrathoracic surgical procedures or invasive diagnostic procedures that required general anesthesia. Specifically, invasive diagnostic procedures included myelocentesis, arthocentesis, skin biopsies and measurements of the cerebrospinal fluid pressure. The surgical procedures included orthopedic surgeries, ocular surgeries, dental procedures, castrations (not including cryptorchid dogs), neurosurgeries and soft tissue surgeries that did not involve excision of full thickness of the abdominal or thoracic wall. All dogs included in the study were listed as an American Society of Anesthesiologists (ASA) physical status 1 or 2. Exclusion criteria were the presence of vomiting, regurgitation or other gastrointestinal symptoms confirmed or observed during the last week, any history of gastrointestinal disease, and the administration of any drug that could alter the motility of the gastrointestinal track, within the last 7 days. All dogs included in the study were assessed with physical examination, complete blood count, serum biochemical profile and radiographic evaluation of the thorax and abdomen. In case of detection of any abnormal finding, the dog was excluded from the study.

All dogs were hospitalized starting from the day before the study to acclimatize and to follow specific instructions regarding fasting. The duration of preoperative fasting was based on previous studies regarding the effect of preoperative fasting on the incidence of reflux [15,21]. All dogs were given half the daily requirements of a commercial canned food the previous evening and 12 h later, they were fed again the same canned food at half the daily requirements. Three hours after their last meal, premedication was administered. All dogs had free access to water up to one hour before premedication. The canned food administered to all dogs was the same during the study and their daily requirements were calculated according to the suggested requirements of the canned food per kg of body weight. In case of a dog exhibiting aversion to the meal or refusing to consume the total amount of food that was given, this dog was excluded from the study.

All dogs were allocated into 9 groups (30 dogs in each group), according to the premedication administered. Premedication consisted of a sedative, dexmedetomidine (Dexdomitor; Elanco, Chalandri, Greece), or acepromazine maleate (Acepromazine; Alfasan, Woerden, The Netherlands), or midazolam (Dormicum; Roche, Marousi, Greece) in combination with an opioid, morphine (Morfina Cloridrato; Molteni, Firenze, Italy) or pethidine (Pethidina Cloridrato, Molteni, Firenze, Italy) or butorphanol (Butomidor; Richter Pharma, Wels, Austria). The combinations and the doses of the drugs administered for premedication are shown in Table 1.

The dogs were not randomly allocated to each group. The decision to keep the study non-randomized was based on the fact that a balanced anesthesia and the welfare of the animals undergoing painful surgical procedures could not be achieved in the groups of midazolam or butorphanol without any additional interference. Thus, dogs undergoing light surgical procedures, such as dental procedures, castrations and other invasive diagnostic procedures that required anesthesia, were assigned to the midazolam or butorphanol groups, whilst dogs undergoing procedures that required a deeper plan of anesthesia were allocated to dexmedetomidine or acepromazine combined with either morphine or pethidine groups. The allocation to groups of dexmedetomidine and acepromazine combined with either morphine or pethidine and to the groups of midazolam and butorphanol was random. To ensure the appropriate levels of analgesia, at the end of the study and before the end of the anesthetic procedure, all dogs received a constant rate infusion (CRI) of fentanyl, and a supplementary local anesthetic–analgesic technique was performed, when possible, to enhance analgesia during recovery. The study was also not blinded, because the measurement of the pH in the esophagus is an objective factor, not influenced by the researcher’s subjectivity.

### 2.2. Experimentation and Anesthetic Management

Three hours after the last meal, the dogs were transferred to the preparation room and premedication was administered according to group allocation. The drugs were mixed in the same syringe and administered intramuscularly (IM). Animals that vomited during premedication were excluded from the study. Approximately 25 min after premedication, induction of anesthesia was performed with propofol (Propofol 1% MCT/LCT; Fresenius Kabi, Hellas, Agia Paraskevi, Greece) intravenously (IV) with a starting dose of 1 mg/kg followed by incremental doses of 1 mg/kg to effect. Intubation was performed when the palpebral reflex was subdued and in the absence of coughing or gagging. Dogs that exhibited coughing or gagging during intubation were excluded from the study. Anesthesia was maintained exclusively with isoflurane in 100% oxygen through a circle breathing system for animals weighing more than 7 kg or through non-rebreathing systems (Jackson-Rees modification of Ayre’s T-piece or Bain) for animals weighing 7 kg or less. Monitoring of the depth of anesthesia was always performed by the same individual. Adequacy of the anesthetic plane was assessed by the absence of the palpebral reflex, the loss of muscle tone (loose jaw) and the maintenance of a steady heart and respiratory rate. Less than 20% changes in heart and respiratory rates during surgical stimulation were ignored. Changes in the heart and respiratory rate above 20% required rescue analgesia with fentanyl (Fentanyl, Janssen-Cilag, Pefki, Greece) bolus at 2 μg/kg IV and the patients were excluded from the study. Standard monitoring also included ECG (lead II), EtCO_2_ (end-tidal partial pressure CO_2_), non-invasive arterial blood pressures and temperature, and values were recorded every 5 min. Heating pads were used to combat hypothermia.

Following intubation, a pH-meter electrode (pH-meter 507, pH electrode 52-00, Crison Instruments, Barcelona, Spain) was inserted into the esophagus about 5 cm above the lower esophageal sphincter (LES). When the electrode was placed into the esophagus, it was attached onto the tracheal tube with a tape, the same as the one used to secure the venous catheter, and was secured in place until the end of the experimentation. To ensure the right distance to the LES, the length up to which the electrode would be inserted, was measured externally from the 10th rib to the incisors [32]. In any case that the electrode was mistakenly inserted into the stomach (detected by a sudden decrease of pH < 4, while the electrode had already inserted at full length) the dog was excluded from the study. The pH was measured for at least 60 min during the surgical procedure or until the end of the procedure and was recorded every 5 min. Gastroesophageal reflux was considered to have occurred when pH values < 4 or >7.5 were detected. The pH-measuring electrode was calibrated in reference values of pH 4 and 7 according to the instructions of the manufacturer. Calibration was performed before any measurement to warrant the precise function of the device.

All animals received carprofen (Rimadyl; Pfizer Inc, Hellas, Neo Psichiko, Greece) at 4 mg/kg IV just after intubation, cefuroxime (Zinacef; GlaxoSmithKline, Chalandri, Greece) at 30 mg/kg IV preemptively and isotonic crystalloids (Lactated Ringer’s) at 10 mL/kg/h, or according to each animal’s specific needs, starting after venous catherization. The use of pre-emptive antibiotic therapy in the invasive diagnostic procedures was recommended by the surgeons and it is not common practice in every case. After preparation of the surgical field, the animal was transported to the operation room with extreme caution to avoid manipulation of the abdomen. Any changes in recumbency were limited to the necessary. In any case that an anesthetic intervention was necessary (that is positive pressure ventilation, intervention for severe bradycardia or hypotension, intervention for supplementary analgesia etc.) the animal was excluded from the study and all necessary measures were implemented.

### 2.3. Post-Evaluation Management

As mentioned above, when all pH measurements were ceased, any required analgesic intervention was instituted to ensure adequate post-operative pain relief. Rescue analgesia with morphine 0.2 mg/kg IM and fentanyl 2 μg/kg IV to effect were administered during recovery as required.

All cases with documented GOR during anesthesia underwent esophageal lavage with NaCl 0.9% (normal saline) at the end of the surgery and received prophylactic treatment for esophagitis. The treatment included metoclopramide (Primperan; Sanofi-Aventis, Kallithea, Greece) at 0.2 mg/kg BID (twice a day) per os, ranitidine (Zantac; GlaxoSmithKline, Chalandri, Greece) at 2 mg/kg BID per os and sucralfate (Peptonorm; Uni-Pharma, Kifisia, Greece) at 30 mg/kg BID per os. Treatment was instituted for 5 days and the owners were encouraged to inform the clinic if they observed any signs of vomiting, hypersalivation, or regurgitation that persisted.

### 2.4. Statistical Analysis

A power analysis revealed that in order to detect a 30% increase in the GOR incidence in either group, with a 1-β error probability 0.8 and α error probability 0.05, a sample size of 30 animals in each group was required. The chi-squared test was used to evaluate associations between qualitative variables and the ANOVA test was used to evaluate any differences between quantitative (continuous) variables.

## 3. Results

### 3.1. Descriptives Results

In this study a total of 270 dogs (111 females and 159 males) were used. Their mean (±standard deviation) age was 4.23 (±2.73) years, their mean Body Weight (BW) was 18.61 (±9.74) kg and the mean dose of propofol (mg/kg) used for induction was 4.29 (±2.57). The duration of pH measurement ranged from 60 min to 105 min (mean 86 ± 16.2) (Table 2).

### 3.2. The Incidence of GOR

Out of the 270 dogs used in this study, 119 (44.1%) experienced a reflux episode. A statistically non-significant difference was found in the incidence of GOR between genders (*p* = 0.173) (Table 3).

The premedication group was associated statistically non-significantly with the incidence of reflux (chi-squared test, *p* = 0.117). However, there was a tendency in group AB to be more likely to develop GOR, while the dogs of group AP showed a tendency to develop GOR less frequently. Groups DM and DP revealed a tendency to present a lower reflux incidence (Table 4), although the incidence of reflux between the two groups did not differ significantly.

In all 119 cases of GOR, the refluxate was acidic, with pH values < 4, except in two cases (1.68%) where the pH of the refluxate was >7.5. (alkaline). Moreover, the pH of the emerged contents in 53 out of the 119 patients which experienced GOR was <2.

### 3.3. Type of Surgical Procedure

Sixty-six out of 270 dogs underwent castration, 35/270 were submitted to dental procedures, 20/270 were submitted to ocular procedures, 82/270 underwent orthopedic surgical procedures, 52/270 underwent non-intrabdominal soft tissue surgery, 6/270 underwent neurosurgery and 9/270 were submitted to invasive diagnostic procedures. Allocation of the different types of surgical procedure are shown in Table 5.

The incidence of reflux differed statistically between dogs that underwent castration compared to dogs that underwent invasive diagnostic procedures (p = 0.009). In particular, dogs that underwent castration were more likely to develop GOR, than dogs that underwent invasive diagnostic procedures. The differences among the other types of surgical procedures were statistically non-significant (Table 6).

### 3.4. Onset and Duration of GOR

The mean onset time of GOR during anesthesia was 19 (±19.3) min. The mean onset of GOR in each group is shown in Table 7. Twenty-nine out of the 119 (24.36%) dogs that experienced a reflux episode, had a pH measurement lower than 4 by the time the pH electrode was inserted into the esophagus, thus implying that a reflux episode had already occurred during premedication or induction. Nine out of 119 (7.56%) dogs with GOR experienced a reflux episode 60 min after insertion of the pH electrode. There was a statistically significant difference between group AB and group DB in relation to the time of onset of GOR during anesthesia (*p* = 0.029). In particular, in group AB, 52.6% of the dogs that experienced reflux developed GOR within 10 min of the beginning of measurements, while 89.5% experienced GOR within the first 30 min. On the other hand, in group DB the onset time of GOR was noted 20 mins after the initiation of measurements. Group DB also differed significantly from group DP (*p* = 0.036) concerning the onset time of reflux. Particularly, in group DP 63% of the dogs that experienced reflux, developed GOR within the first 10 min. The duration of GOR ranged from 5 min to the end of the surgical procedure (105 min). The mean duration of reflux episodes was 53.7 (±28) min. The mean duration of GOR in each group is shown in Table 7. In group AM the duration of reflux was significantly longer when compared with group MP (*p* = 0.042).

### 3.5. Age, BW, Propofol Dose

Some groups differed significantly regarding the age. Particularly in groups AM and AP the mean age was 5.2 (±3.43) and 6.03 (± 3.43) respectively, while in the rest of the groups the mean age was lower. However, this heterogeneity in age distribution did not affect the incidence of reflux (p = 0.071). There were statistically non-significant differences in BW among groups. The mean dose of propofol differed significantly among groups especially in midazolam and acepromazine groups. The highest mean dose (6.94 ± 2.74) was observed in the MM group. However, the difference in propofol dosage among groups did not affect the incidence of reflux among the groups (p = 0.219).

### 3.6. Type of Recumbency

The differences among groups regarding the type of recumbency were statistically non-significant (p = 0.235). Out of the 270 dogs, 12 were placed in dorsal, 148 in lateral and 110 in sternal recumbency.

## 4. Discussion

In the present study 270 dogs underwent various types of surgical procedures under general anesthesia. Of the 270 dogs 119 (44.1%) experienced a reflux episode during anesthesia. The incidence of reflux reported in this study is in accordance with the incidence of reflux reported in veterinary literature. In particular, in veterinary literature, general anesthesia in dogs that had premedication which included an opioid resulted in an incidence of reflux ranging from 36.17% to 56.6% [19,26,27,28]. When anesthesia did not include any opioids in premedication, the incidence of GOR was recorded at 16.3% and reached up to 17.4 % in intra-abdominal surgeries [15,16]. Interestingly, the only opioid used in the two latter studies was pethidine, and was used as a single premedication agent in one group. The lower incidence of reflux observed in the absence of opioids has also been reported in a study performed on dogs undergoing ovariohysterectomies, where reflux was recorded at 13.3%, despite the fact that all dogs underwent an intra-abdominal surgical procedure [14,33]. Our results considered in conjunction with the results described in the literature indicate that the inclusion of opioids in premedication increases the incidence of reflux in anesthetized dogs.

In two out of 119 cases of GOR (1.68%) the emerged content had a pH > 7.5 characterized as alkaline, while in all other cases the pH of the refluxate was acidic (<4). The percentage of alkaline reflux observed in this study was much lower than that described in the literature. In particular, the percentage of alkaline pH recorded in several studies ranged from 7.5% to 10.3% [15,16,26]. In our study, 53 out of the 119 patients (44.5%) that experienced GOR had pH of the refluxate < 2, which was unexpected taking into account that a 3-hour fasting period would at least lower the acidity of the emerged contents, as mentioned in previous studies [15,21,34]. Nonetheless, in a study comparing different fasting times (2–4 h and 12–18 h) statistically non-significant differences were found concerning the acidity of the refluxate [2]. Moreover, a study investigating preanesthetic fasting in dogs, reported a significantly lower pH of the emerged content in >24 h fasting time, but non-significant difference was detected amongst 2–4 h and 12–18 h fasting on the pH of the refluxate [15].

In our study, in group AB the incidence of GOR was higher, although statistically non-significant, in contrast with group AP in which the lower incidence of reflux was detected, with a tendency in both groups for statistically significant differences. In particular, 66.7% of the patients in group AB developed GOR, while in group AP 26.7% of the dogs experienced GOR. The use of acepromazine has been found to decrease the pressure of the Lower Esophageal Sphincter (LES), through the inhibition of 5-hydrotriptamine [3]. Moreover, the effect of acepromazine is dependent on the administration route. For example, intravenous administration of acepromazine produced a decrease of LES pressure up to 61% 15 min after administration, while intramuscular administration resulted in delay in its action on the LES up to 50 min in [3,17,35]. In a study by Anagnostou et al. seven female dogs were anesthetized three times each, with acepromazine, thiopentone and halothane, and esophageal pH was monitored for an hour. A reflux episode was reported in only one dog [31]. In the present study, the decrease in LES pressure, caused by the administration of acepromazine, could have resulted in a higher incidence of reflux, however in group AP the incidence of recorded GOR was the lowest. Our results were in agreement with the literature, and suggest that the use of acepromazine does not increase the incidence of reflux. However, the addition of opioids in premedication may alter the expected results.

The effect of pethidine on the LES pressure is not fully clarified. In one study, pethidine was reported to reduce the LES pressure [36], while in a second study, pethidine has been found to produce a phasic increase in LES tone [3]. When pethidine was used as the single premedication agent, the incidence of reflux appeared to be low (10%) [15]. Likewise, the administration of pethidine combined with acepromazine did not increase the incidence of GOR when compared with the administration of pethidine used alone in premedication [28]. Our results were in accordance with the above-mentioned findings, as the combination of acepromazine and pethidine (group AP) resulted in the lowest incidence of GOR. On the other the hand, the use of opioids in premedication, particularly morphine and methadone, has been associated with a higher incidence of reflux. More specifically, 63% of the dogs that experienced regurgitation during anesthesia, had received morphine in premedication and only in one case (3.7%) butorphanol had been administered in premedication [18]. The use of morphine reduces LES pressure and increases the incidence of reflux during anesthesia [27,36]. We expected to observe a higher incidence of GOR in groups with morphine, as reported in other studies. However, this was not noticed, as the higher incidence of reflux was detected in groups AB and DB, where butorphanol was the opioid administered. A possible explanation for the lower incidence of GOR in morphine groups observed in this study, may be the lower dosage of morphine used (0.1 mg/kg) in contrast to the doses used in other studies [27,28].

The effect of butorphanol on the LES and on the incidence of reflux in anesthetized dogs has not been widely evaluated. A study by McFadzean et al. revealed that the use of butorphanol in premedication did not increase the incidence of reflux when compared to methadone. In particular, 2/10 dogs in the butorphanol group and 1/10 in the methadone group experienced GOR [37]. In another study 21 out of 24 dogs that were administered either butorphanol or methadone in premedication experienced a reflux episode. Although the exact number of dogs premedicated with butorphanol and refluxed was not clearly stated, the reflux episodes were increased and no statistically significant difference between butorphanol and methadone on the incidence of reflux was detected [38]. In our study, the use of butorphanol in combination with acepromazine or dexmedetomidine increased the incidence of GOR with the AB group reaching an incidence of 66.7% of reflux and DB group 53.3%. However, no statistically significant differences were detected. A larger sample in each group may have resulted in a statistically significant outcome.

The stimulation of a-2 adrenergic receptors has been found to increase contraction of the LES in dogs and humans [3,39]. In humans, the use of dexmedetomidine has been known to slightly decrease the LES pressure, however the reduction of LES pressure observed was not capable of causing GOR [40]. When dogs were premedicated with a combination of dexmedetomidine and hydromorphone, GOR was detected with a significantly lower incidence in comparison to premedication with hydromorphone alone [41]. In our study, the incidence of reflux in groups DP and DM (36.7% in both groups) was lower, though not significantly, than the rest of the groups (>43.3%), except for group AP (26.7%) in which the lowest, although non-significant, incidence of GOR was noted. These results were in agreement with previous studies supporting the lower incidence of reflux when dexmedetomidine or other a-2 agonists were administered with or without the addition of opioids [3,39,40,41]. On the contrary, a study investigating regurgitation in anesthetized dogs reported an increased incidence of regurgitation following premedication with medetomidine alone when compared with the combination of acepromazine and an opioid [42]. Furthermore, the administration of xylazine resulted in a 77% reduction on LES pressure [3]. Thus, the effect of a-2 agonists on the incidence of GOR in dogs, needs to be further investigated.

To our knowledge, the effect of midazolam on the incidence of reflux has not been evaluated in dogs. However in rabbits, the use of midazolam has produced a relaxation of the LES [43]. Diazepam, another benzodiazepine, has been found to reduce LES pressure in dogs, nonetheless whether this pressure reduction is capable of producing GOR is unknown [17]. In dogs premedicated with diazepam solely, the incidence of GOR remained low [15]. In our study, the combination of midazolam with opioids did not produce statistically different effects when compared with dexmedetomidine or acepromazine. The development of reflux in these groups remains within the reported percentages in the literature, although higher than those reported for diazepam [15,19,25,26,27,28]. A reasonable explanation is that the addition of opioids may increase the incidence of reflux [18,27], or that the higher propofol dosage in midazolam groups may have contributed to an increased incidence of reflux.

In our study, the dose of propofol needed to ensure intubation without coughing ranged from 1 up to 20 mg/kg. The higher mean dosage of propofol was observed in the midazolam groups. All midazolam groups (MM, MP, MB) differed significantly from the acepromazine and dexmedetomidine groups in regard to the dosage of propofol. Nonetheless, these differences did not reflect an equivalent significant increase in the incidence of reflux. In the literature, the use of propofol has been shown to produce a decrease in LES barrier pressure (<10 mmHg) in all but one animal (1/24 dogs) [44]. In humans, when barrier pressure drops below 10 mmHg, the incidence of reflux is increased [45]. Although, the use of propofol has been found to reduce LES pressure and may result in a subsequent increase on the incidence of reflux in dogs [44,46], in our study that was not observed.

This study revealed that there was a significant difference in the time of onset of reflux in groups AB and DB. In particular, 52.6% of the GOR episodes in group AB, were recorded during the first ten minutes from the initiation of pH measurement, while in group DB 50% of the dogs refluxed within 30 min after commencement of the measurements (p = 0.029). A similar significant difference was also observed between groups DB and DP (p = 0.036). Specifically, in group DP 63% of the GOR episodes were recorded within ten minutes from the beginning of the measurement, while in group DB 50% of the dogs refluxed within 30 min from the beginning of measurements, as mentioned previously. A similar outcome has been reported in a previous study, where more than 50% of the GOR episodes were observed within 10 min after the insertion of the pH-meter [46]. Panti et al. noted that all dogs refluxed within 60 min from the initiation of the pH measurement [19], while there is only one report of a GOR episode that developed after 60 min of anesthesia [15]. Likewise, in our study only 7.56% (9/119) of reflux episodes were recorded 60 min after insertion of the pH-meter. Our findings were in accordance with the literature, suggesting that GOR in anesthetized dogs develops early.

Duration of GOR ranged from 5 to 105 min (which was considered the end of the measurement period). The mean duration was 53.7 min. Duration of GOR was significantly longer in group AM when compared with group MP (p = 0.042). Specifically, in 69.2% of the dogs with GOR in group AM, the duration of reflux was >65 min, while in group MP in 71.4% of the dogs with GOR the max time did not exceed 55 min. The mean duration of GOR in two similar studies was reported from 56 to 101 and 87 to 100.8 min [25,28]. In both of these studies opioids had been used in premedication. On the other hand, in studies where no opioids were used the reported mean duration of reflux was lower. For example, in a study by Galatos et al., the only opioid used in premedication was pethidine, in one group, and the mean duration of GOR was reported at 47.8 min [15]. In another study without the administration of opioids, the mean duration of reflux was 55.8 and 42.8 min in the two groups studied [46]. Favarato et al., reported 4 dogs with reflux, in two of which the duration of reflux lasted 18 min and 4 min. No information was given for the other two dogs that refluxed [14]. None of the dogs was premedicated with opioids; however, the sample size was too small to safely draw any conclusions.

In our study the age was not equally distributed among groups. However, this heterogeneity did not affect the incidence of reflux among groups (p = 0.071). In veterinary literature the age is not reported to influence the incidence of reflux, only a tendency to an increased incidence of GOR as age grows has been observed, nonetheless this observation was not statistically significant [16]. Gender did not affect the incidence of reflux, as there were no significant differences in the incidence of GOR between male and female dogs, an outcome that confirms previous studies [16,47]. There were no differences in the distribution of the BW of the dogs used in this study, nor there was any evidence that BW affected the incidence of reflux. Our results were in contrast with a study performed on dogs, which revealed that dogs heavier than 40 kg develop GOR more often that smaller dogs [18]; however, the maximum BW included in our study was 50 kg. Our results were in accordance with two studies performed earlier, which also revealed no correlation between BW and reflux in anesthetized dogs [15,16].

In veterinary medicine the type of surgery is correlated with the incidence of reflux. Intra-abdominal surgical procedures are related to a higher incidence of reflux due to increases in intra-abdominal pressure. Uterine surgery has been described as the type of surgery with the higher incidence of GOR [16]. However, other studies have demonstrated a lower incidence of GOR during intra-abdominal surgeries. For example, in a study of 30 dogs undergoing elective ovariohysterectomy only 4 dogs (13.3%) developed GOR during the procedure [14]. Similar outcomes have been observed in another study with dogs undergoing ovariohysterectomies, and only 18.03% of them developed GOR [31]. The incidence of reflux recorded in the two aforementioned studies wass lower compared to our results, despite the fact that none of our dogs underwent any intra-abdominal or intra-thoracic surgery. It is interesting to note that in all previously mentioned intra-abdominal surgeries, no opioids had been administered. This may have been a possible explanation of the low incidence of reflux recorded. In our study, the highest incidence of reflux was observed in castrations, where 59.1% of the dogs developed GOR and the lowest in invasive diagnostic procedures where no dog refluxed. The incidence of reflux in castrations and in diagnostic procedure was significantly different (p = 0.009). On the other hand, orthopedic surgeries revealed a 36.6% of GOR (30/82 dogs), with a non- significant difference compared with the other surgical procedures. In veterinary literature, orthopedic surgeries are correlated with a higher risk of regurgitation and reflux [18,38,47], nevertheless our findings did not support this correlation. This may be explained by the low doses of opioids used in premedication or by the careful manipulation and minimum changes in recumbency during the procedures. A recent study has demonstrated the high incidence of regurgitation in diagnostic imaging procedures. These results have been potentially explained by changes in the anesthetic depth, handling and changing recumbency [42]. In our study, no evidence of reflux in any of the dogs was observed in invasive diagnostic procedures. However, the sample size was small, with only nine dogs being included in such procedures. On the other hand, neurosurgeries also included a small sample size with only 6 dogs, however non-significant differences were detected when compared to the other types of surgeries.

The present study revealed that the type of recumbency did not influence the incidence of reflux. No statistically significant differences were found among lateral, sternal or dorsal recumbency in the incidence of GOR. These results were in accordance with previous studies wherein the type of recumbency. or even the 8^0^ head-down or head-up positioning, had no effect on the incidence of reflux [16,48].

One limitation of the study was the non-randomized selection of the groups, which may lead to biased results. However, the investigator did not select upon preference the allocation to groups. The groups were divided into two categories, the midazolam-butorphanol groups (MM, MP, MB, AB, DB), where the less painful procedures were included, and the dexmedetomidine—acepromazine—morphine—pethidine groups (DM, DP, AP, AM) which comprised the more painful procedures. The allocation within the two categories was randomized. Another limitation of the study was the non-even allocation of the surgical procedures within the groups. For example, the invasive diagnostic procedures and the neurosurgeries were performed in only nine and six dogs respectively, while 82 dogs underwent orthopedic surgeries, and that could have interfered with the final outcomes, especially in procedures with small sample size.

## 5. Conclusions

In this study the incidence of reflux recorded in anesthetized dogs was within the limits reported in the veterinary literature. There were no significant differences among groups concerning the incidence of GOR, and in the majority of the cases the refluxed content was acidic, as described in previous studies. Furthermore, the age, gender, body weight and type of recumbency were not associated with increased episodes of GOR. Nonetheless, the addition of opioids in premedication was related with a higher incidence of GOR, although non-significant, in comparison to the premedication without opioids as reported in the literature. In the present study, the use of morphine in combination with either dexmedetomidine, acepromazine or midazolam did not significantly increase the incidence of reflux when compared with pethidine or butorphanol. Orthopedic surgeries were not associated with a significantly higher risk of GOR. On the other hand, the incidence of reflux was statistically higher in castrations when compared with invasive diagnostic procedures.

## Figures and Tables

**Table 1:** Premedication drugs and the doses administered in each of 9 experimental group of dogs submitted to esophageal pH measurement during anesthesia for non-intrabdominal, non-intrathoracic procedures.

Group	Premedication	Doses
DM	Dexmedetomidine	150 μg/m^2^
	Morphine	0.1 mg/kg
DP	Dexmedetomidine	150 μg/m^2^
	Pethidine	2 mg/kg
DB	Dexmedetomidine	150 μg/m^2^
	Butorphanol	0.1 mg/kg
AM	Acepromazine	0.05 mg/kg
	Morphine	0.1 mg/kg
AP	Acepromazine	0.05 mg/kg
	Pethidine	2 mg/kg
AB	Acepromazine	0.05 mg/kg
	Butorphanol	0.1 mg/kg
MM	Midazolam	0.2 mg/kg
	Morphine	0.1 mg/kg
MP	Midazolam	0.2 mg/kg
	Pethidine	2 mg/kg
MB	Midazolam	0.2 mg/kg
	Butorphanol	0.1 mg/kg

**Table 2:** Descriptive results (mean and standard deviation) for age, body weight, propofol dosage, duration of measurements, duration of GOR and onset time of GOR in dogs submitted to esophageal pH-measurements during anesthesia for non-intrabdominal, non-intrathoracic procedures. (n= 270).

Variables	Mean (±Std)
Age (years)	4.23 (±2.73)
Body Weight (kg)	18.61 (±9.74)
Propofol (mg/kg)	4.29 (±2.57)
Duration of measurements (min)	86 (±16.2)
Duration of GOR (min)	53.7 (±28)
Time of onset of GOR (min)	19 (±19.3)

Std: standard deviation, N: total number of dogs included in the study.

**Table 3:** The incidence of GOR between genders in dogs submitted to esophageal pH-measurement during anesthesia for non-intrabdominal, non-intrathoracic procedures.

Gender	n^1^	GOR	
		YES	NO
Female	111	43 (38.7%)	68 (61.3%)
Male	159	76 (47.8%)	83 (52.2%)
Total	270	119 (44.1%)	151 (55.9%)

^1^ n = number of dogs.

**Table 4:** The incidence of GOR within the premedication groups in dogs submitted to esophageal pH-measurement during anesthesia for non-intrabdominal, non-intrathoracic procedures. (n = 30 dogs in each group).

Group		Reflux		Total (%)
		No (%)	Yes (%)	
AB	Count	10 ^a^ (33.3%)	20 ^b^ (66.7%)	30 (100%)
	Residual	−6.8	6.8	
	Std. Residual	−1.7	1.9	
	Adjusted Residual	−2.6	2.6	
AM	Count	17 ^a^ (56.7%)	13 ^a^ (43.3%)	30 (100%)
	Std. Residual	0.1	−0.1	
	Adjusted Residual	0.1	−0.1	
AP	Count	22 ^a^ (73.3%)	8 ^b^ (26.7%)	30 (100%)
	Std. Residual	1.3	−1.4	
	Adjusted Residual	2.0	−2.0	
DB	Count	14 ^a^ (46.7%)	16 ^a^ (53.3%)	30 (100%)
	Std. Residual	−0.7	0.8	
	Adjusted Residual	−1.1	1.1	
DM	Count	19 ^a^ (63.3%)	11 ^a^ (36.7%)	30 (100%)
	Std. Residual	0.5	−0.6	
	Adjusted Residual	0.9	−0.9	
DP	Count	19 ^a^ (63.3%)	11 ^a^ (36.7%)	30 (100%)
	Std. Residual	0.5	−0.6	
	Adjusted Residual	0.9	−0.9	
MB	Count	17 ^a^ (56.7%)	13 ^a^ (43.3%)	30 (100%)
	Std. Residual	0.1	−0.1	
	Adjusted Residual	0.1	−0.1	
MM	Count	18 ^a^ (60.0%)	12 ^a^ (40.0%)	30 (100%)
	Std. Residual	0.3	−0.3	
	Adjusted Residual	0.5	−0.5	
MP	Count	15 ^a^ (50.0%)	15 ^a^ (50.0%)	30 (100%)
	Std. Residual	−0.4	0.5	
	Adjusted Residual	−0.7	0.7	
Total	Count	151 (55.9%)	119 (44.1)	270 (100%)

^a^ The incidence of reflux did not differ statistically within groups; ^b^ The incidence of reflux presented a tendency in statistical difference (chi-square test, std residual 1.9) within groups. Each subscript letter denotes a subset of reflux categories whose column proportions do not differ significantly from each other at the 0.05 level.

**Table 5:** The number of surgical procedures allocated to groups, in dogs submitted to esophageal pH-measurement during anesthesia for non-intrabdominal, non-intrathoracic procedures. (n = 30 dogs in each group, 270 dogs in total).

Groups	Type of Surgical Procedure						
	Castration	Ortho ^1^	Soft Tissue ^2^	Dental ^3^	Eye Surgery	Neurosurgery	Diagnostic ^4^
DM (30)	1	22	4	1	0	2	0
DP (30)	2	15	8	4	0	1	0
DB (30)	8	11	6	1	4	0	0
AM (30)	0	15	12	2	0	1	0
AP (30)	4	8	11	3	2	2	0
AB (30)	12	4	7	3	4	0	0
MM (30)	13	4	2	2	2	0	7
MP (30)	13	2	2	8	5	0	0
MB (30)	13	1	0	11	3	0	2
**Total (270)**	**66**	**82**	**52**	**35**	**20**	**6**	**9**

^1^ Ortho = orthopedic surgery, ^2^ Soft Tissue = soft tissue surgery, ^3^ Dental = dental procedure, ^4^ Diagnostic = invasive diagnostic procedures.

**Table 6:** The incidence of GOR within the surgical procedures in dogs submitted to esophageal pH-measurement during anesthesia for non-intrabdominal, non-intrathoracic procedures.

Surgical Procedures	GOR	
	YES	NO
Castration ^a,b^	39 (59.1%)	27 (40.9%)
Dental procedures ^a^	14 (40%)	21 (60%)
Invasive diagnostic procedures ^a,b^	0 (0%)	9 (100%)
Eye surgery ^a^	11 (55%)	9 (45%)
Neurosurgery ^a^	2 (33.3%)	4 (66.7%)
Orthopedic surgery ^a^	30 (36.6%)	52 (63.4%)
Soft tissue surgery ^a^	23 (55.8%)	29 (44.2%)

^a^ The incidence of GOR did not differ statistically among the surgical procedures, ^b^ the incidence of GOR differed significantly among the surgical procedure.

**Table 7:** The mean (±std) duration of GOR and onset time in each of the 9 experimental groups of dogs submitted to esophageal pH-measurement during anesthesia for non-intrabdominal, non-intrathoracic procedures. (n = -30 dogs in each group, 270 dogs in total).

Groups	Duration of GOR	Onset Time of GOR
	Mean (±std)	Mean (±std)
AB	60.7 (±33.3)	13.2 (±16.9)
AM	63.8 (±33.4)	13.4 (± 10.8)
AP	46.2 (±34.0)	21.8 (±23.5)
DB	51.2 (±22.0)	27.5 (±22.2)
DM	56.3 (±35.7)	24.0 (±27.9)
DP	61.5 (29.5)	11.0 (±15.0)
MB	53.8 (±18.6)	20.0 (±20.4)
MM	45.0 (±20.5)	24.5 (±18.6)
MP	42.0 (±21.0)	17.3 (±15.6)

## Data Availability

Data presented in this study are available on request from the corresponding author.
